# Bariatric Surgery Is Associated with Alcohol-Related Liver Disease and Psychiatric Disorders Associated with AUD

**DOI:** 10.1007/s11695-023-06490-w

**Published:** 2023-03-07

**Authors:** Edilmar Alvarado-Tapias, David Marti-Aguado, Kevin Kennedy, Carlos Fernández-Carrillo, Meritxell Ventura-Cots, Dalia Morales-Arraez, Stephen R. Atkinson, Ana Clemente-Sanchez, Josepmaria Argemi, Ramon Bataller

**Affiliations:** 1 Center for Liver Diseases, Division of Gastroenterology, Hepatology and Nutrition, Department of Medicine, University of Pittsburgh Medical Center, 201.19 Kaufmann Medical Building, 3471 Fifth Avenue, Pittsburgh, PA 15213 USA; 2 Department of Gastroenterology, Hospital Santa Creu I Sant Pau, Institut de Recerca Sant Pau, Universidad Autónoma de Barcelona, Barcelona, Spain; 3 Centro de Investigación Biomédica en Red de Enfermedades Hepáticas Y Digestivas (CIBERehd), Instituto de Salud Carlos III, Madrid, Spain; 4 Digestive Disease Department, Clinic University Hospital, Biomedical Research Institute INCLIVA, Valencia, Spain; 5 Kansas City, MO USA; 6 Liver Unit, Department of Gastroenterology and Hepatology, Hospital Universitario Puerta de Hierro-Majadahonda, IDIPHISA, Madrid, Spain; 7 Liver Unit, Hospital Universitari Vall d’Hebron, Vall d’Hebron Institute of Research (VHIR), Universitat Autònoma de Barcelona, Barcelona, Spain; 8 Department of Gastroenterology, Hospital Universitario de Canarias, Tenerife, Spain; 9 Department of Metabolism, Digestion and Reproduction, Faculty of Medicine, Imperial College London, London, UK; 10 Liver Unit, Department of Digestive Diseases, Hospital General Universitario Gregorio Marañón, Madrid, Spain; 11 Liver Unit, Clínica Universidad de Navarra (CUN), Hepatology Program, Centro de Investigacion Medica Aplicada (CIMA), Instituto de Investigación de Navarra (IdisNA), University of Navarra, Pamplona, Spain; 12 Department of Medicine, Faculty of Medicine and Health Sciences, University of Barcelona, Barcelona, Spain

**Keywords:** Bariatric surgery, Alcohol use disorder, Alcohol-related liver disease, Alcohol-related mental disorder, Vitamin D

## Abstract

**Background/Aims:**

Bariatric surgery can increase the risk of addictive disorders and nutritional deficiencies. The aim of this study was to evaluate the association between bariatric surgery and alcohol use disorder (AUD), alcohol-related liver disease (ALD), and psychiatric disorders associated with AUD. The impact of vitamin D deficiency in these associations was also investigated.

**Methods:**

A cross-sectional study was performed using the National Inpatient Sample database and its ICD-9 codes information. Diagnostic and comorbidity data from hospital discharges were obtained from patients with bariatric surgery and other abdominal surgeries between 2005 and 2015. The two groups were then compared for alcohol-related outcomes after propensity-score matching.

**Results:**

The final study cohort included 537,757 patients with bariatric surgery and 537,757 with other abdominal surgeries. The bariatric surgery group had an increased risk of AUD [odds ratio (OR): 1.90; 95% CI: 1.85–1.95], ALD [OR: 1.29; 95% CI: 1.22–1.37], cirrhosis [OR, 1.39; 95% CI: 1.37–1.42], and psychiatric disorders associated with AUD [OR, 3.59; 95% CI: 3.37–3.84]. Vitamin D deficiency did not impact in the association between bariatric surgery and AUD, ALD, or psychiatric disorders associated with AUD.

**Conclusions:**

Bariatric surgery is associated with an increased prevalence of AUD, ALD, and psychiatric disorders associated with AUD. These associations appear to be independent from vitamin D deficiency.

**Graphical Abstract:**

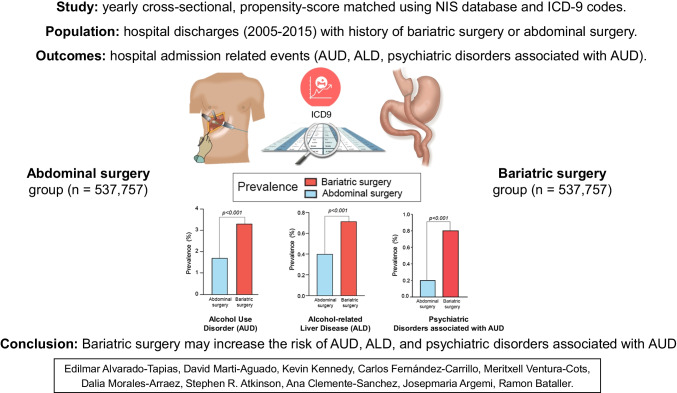

**Supplementary Information:**

The online version contains supplementary material available at 10.1007/s11695-023-06490-w.

## Introduction

Despite the benefits of bariatric surgery among patients with clinically severe obesity, this procedure can be associated with deleterious health effects, particularly those associated with alcohol use. Data from large cohorts suggest that metabolic surgery can increase the risk of alcohol use disorder (AUD) [1–5]. For this reason, US clinical guidelines consider current alcohol or drug use disorders as exclusion criteria for bariatric procedures [6]. However, it is not clear whether a previous metabolic surgery increases the risk of alcohol-related liver disease (ALD) and liver-related events.

Only few data from small samples have investigated the association between bariatric surgery and ALD [7–9]. Mendoza et al. reported that more than 50% of postoperative decompensated liver events were attributable to alcohol use [8]. Among the mechanisms involved in this association, the following have been proposed: (1) changes in alcohol pharmacokinetic, with a rapidly attained maximum blood alcohol concentration (BAC) disproportionately high in relation to the dose consumed [10–13], and (2) changes in hormonal secretion, with neuro-hormonal shifts after bariatric surgery determining changes in reward processing and dopamine signaling [14]. (3) Neurobiological findings in preclinical models with Roux-en-Y gastric bypass (RYGB) have shown that changes in leptin, ghrelin, and GLP-1 secretion could play a major role in the regulation of food and alcohol intake [14–18].

A further potential mechanism linking bariatric surgery with liver disease is vitamin D deficiency. RYGB is associated with a deficit in the absorption of vitamin D [3, 19], and low levels of vitamin D may predispose patients to certain mental health disorders such as depression and addictions, including AUD [20, 21]. In this line, fewer sunlight hours are associated with a higher prevalence of AUD and alcohol-related cirrhosis [22]. In addition, in patients with cirrhosis and/or ALD, vitamin D deficiency is associated, through different mechanisms, with disease severity and mortality [16–19].

Based on this evidence, the hypothesis of the present study was that a prior history of bariatric surgery could increase the risk of developing ALD and psychiatric disorders associated with AUD and that vitamin D deficiency could mediate the alcohol-associated outcomes following this procedure.

## Methods

### Data Source for the Study Population

A cross-sectional observational study was performed using the National Inpatient Sample (NIS) database, which is part of the Healthcare Cost and Utilization Project sponsored by the Agency for Healthcare Research and Quality.

The NIS is a well-characterized database of hospital inpatient stays derived from clinical data submitted by hospitals to statewide data collection organizations across the USA. It contains a wide range of hospital admission information, of which it was considered for this study the following: demographic characteristics, discharge diagnosis (outcomes of interest), and comorbidities (past medical history). Data are collected from a systematic sampling of 20% discharges from all hospitals and stratified by center, census division, ownership status, urban vs rural location, teaching status, bed size, and patient diagnosis-related group, and admission month [23]. This weighting scale provides a sample of hospital discharges from all hospitals nationally. The International Classification of Diseases system (ICD-9-CM codes) was used to identify bariatric surgeries, abdominal surgeries, and outcomes of interest, as described elsewhere [24]. Table 1S summarizes the ICD-9 codes used to define the variables of interest in this study.

**Fig. 1:**
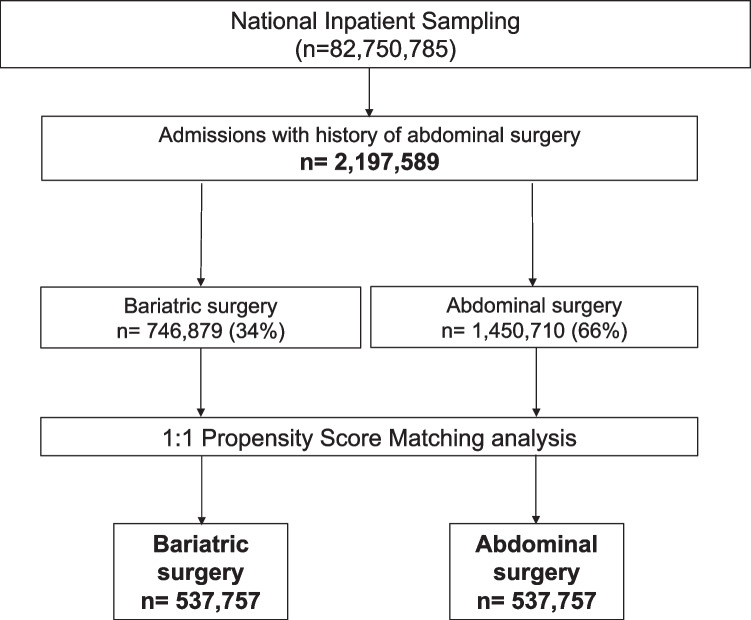
Flow chart showing patient selection from the NIS database. ICD-9-CM: International Classification of Diseases, Ninth Revision—Clinical Modification. *Criteria for selection of patients with “Bariatric surgery” (ICD-9-CM): Bariatric surgery v45.86, 44.68, 43.82, 44.39. †Criteria for selection of “Abdominal surgery” patients (ICD-9-CM): Appendectomy 47.0, incidental appendectomy 47.1, laparoscopic appendectomy 47.01, laparoscopic incidental appendectomy 47.1, cholecystectomy 51.22, laparoscopic cholecystectomy 51.23

Patients admitted during the years 2005–2015, with medical history of bariatric surgery and other abdominal surgeries were included in the study. Cases with abdominal or bariatric surgery were identified using their specific ICD-9 codes (Table 1S) among the secondary discharge diagnosis. The “bariatric surgery group” included sleeve gastrectomy, adjustable gastric band, and RYGB. The “abdominal surgery group” included appendectomy and cholecystectomy. From eligible population, cases with any outcome of interest among the pre-surgical comorbidities were excluded (any code of AUD, liver diseases, psychiatric disorders associated with AUD, and/or vitamin D deficiency). To balance the presence of risk factors between groups, a propensity score match was performed by confounding variables present prior to surgery. Patient matching was performed considering potential factors related to liver disease and mental disorder: age, sex, race, obesity, diabetes, arterial hypertension, renal failure, weight loss, anemia, and primary payer. From finally selected population, study outcomes were identified from the main primary discharge diagnosis. The study design is graphically summarized in Fig. 1S.

### Inclusion and Exclusion Criteria

All hospital discharges between 2005 and 2015 from the NIS database were reviewed for eligibility criteria. Cases with bariatric and other abdominal surgeries were identified with ICD-9 codes and selected for possible inclusion. From the eligible population, cases with the following codes in the pre-surgical comorbidities’ files were excluded: AUD, liver disease, psychiatric disorders associated with AUD, vitamin D deficiency, digestive malignant neoplasm, children, and pregnancy. Viral liver disease was not excluded since the prevalence of this condition in both study groups was extremely low (< 0.3%).

### Definition of Study Outcomes and Variables

The main study outcomes were AUD, ALD, and psychiatric disorders associated with AUD. The three of them are composite outcomes defined by different ICD-9 codes (Table 1S). Liver disease and psychiatric disorder outcomes were composed by specific codes related to their disease and alcohol. For instance, AUD was defined as any code related to alcohol abuse, alcohol dependence syndrome, and/or alcoholism according to the DSM-5 manual. Recreational drug use was considered an addictive disorder with a proper ICD-9 code. ALD was defined as the presence of any of the following diagnoses: alcoholic fatty liver, acute alcoholic hepatitis, cirrhosis of the liver with alcoholism, and/or alcoholic liver disease. Obesity-associated liver disease was defined with the specific code “nonalcoholic fatty liver disease.” Psychiatric disorders associated with AUD were defined with any of the following codes: alcohol abuse with psychotic and panic disorders, depression, stress, anxiety, and bipolar syndrome [25]. Depression without AUD was also considered a mental disorder with a proper ICD-9 code. All these conditions being studied were not present prior to surgery.

The main aim of the study was to compare the prevalence of ALD and psychiatric disorders associated with AUD in the bariatric surgery group vs abdominal surgery group. Demographic characteristics and clinical data were collected. The influence of vitamin D deficiency was also determined.

#### Ethical Considerations

The study was performed in accordance with the Declaration of Helsinki and good clinical practice guidelines. The local institutional review board (University of Pittsburgh Institutional Revision Board) approved the study protocol in May 14, 2019 (STUDY19010143).

### Statistical Analysis

For continuous and categorical variables, data were shown as means ± standard deviation (SD) or *n* (%). Prevalence rates for the main outcome variables were shown as the total prevalence (%) and as overall and yearly fold-changes. The fold-change refers to the changes in prevalence in both groups, considering fold-increase changes for each outcome in the bariatric surgery group vs abdominal surgery group.

To account for the non-random selection of groups, a 1:1 propensity score was performed, matching patients by potential factors related to liver disease and mental disorder. This matching was performed prior to surgery based on the following variables: age, sex, race, obesity, diabetes, hypertension, renal failure, weight loss, anemia, and primary payer. The decision to calculate a propensity score was based on a well-validated method to balance the large number of covariates across the two groups [26, 27]. In this study, the propensity score was defined as the conditional probability of being in the bariatric surgery group in a multivariable logistic regression model. A caliper width of 0.2 times the SD of the propensity score on the logit scale was used and determined the balance of matched variables by using the standardized difference, with values < 10 being the threshold for a quality match [27]. After matching, the outcomes of interest were compared between bariatric surgery and abdominal surgery using conditional stratified logistic regression, accounting for matched pairs as random effects. The results are reported as odds ratios (OR) with 95% confidence intervals (CI). Was used the SAS 9.4 software (SAS Institute, Inc.; Cary, NC; USA) for all analyses.

## Results

### Baseline Characteristics

**Fig. 2:**
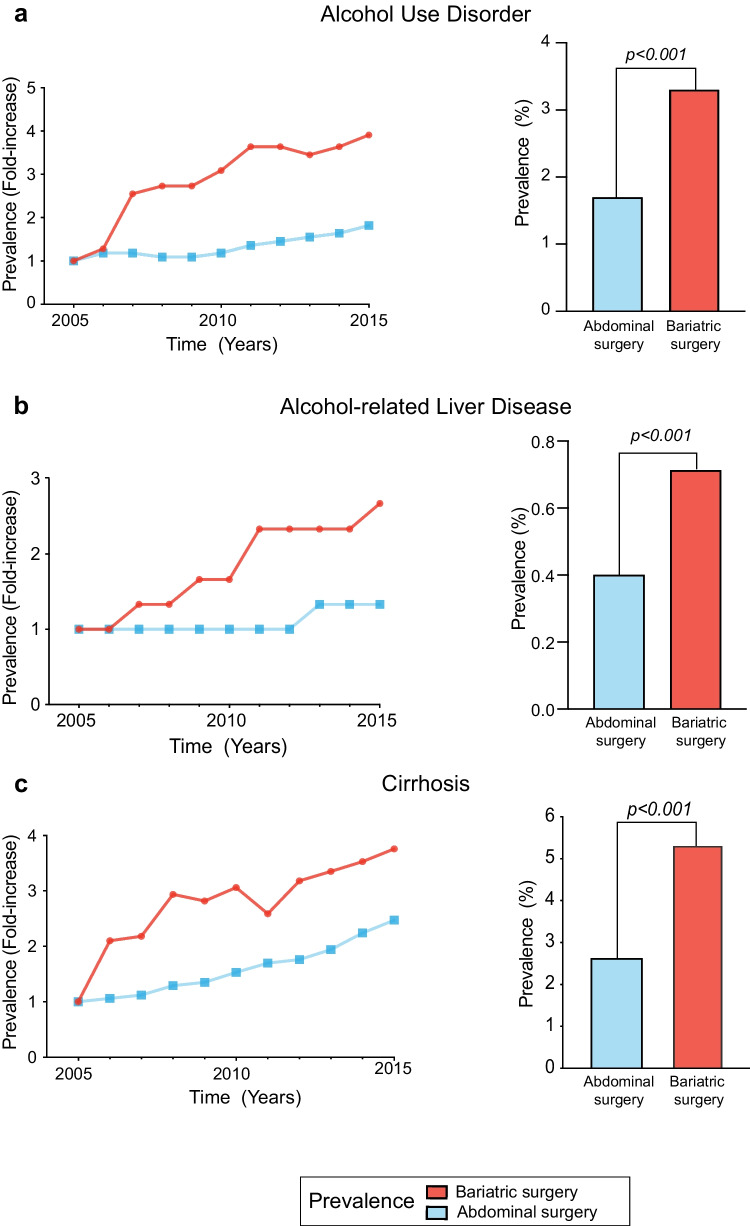
Impact of bariatric surgery on the incidence of alcohol use disorder, alcohol-related liver disease, and liver cirrhosis from 2005 to 2015. Results are expressed as annual fold changes (left side) and overall prevalence rate (right side) during the study period (2005 to 2015)**. a** Prevalence of alcohol use disorder;** b** prevalence of alcohol-related liver disease; and **c** prevalence of liver cirrhosis in the bariatric surgery and abdominal surgery groups

A total of 82,750,785 hospital discharges were evaluated. Of these, 2,197,589 patients met the study inclusion criteria; 34% had undergone bariatric surgery (bariatric surgery group) and 66% other surgeries (abdominal surgery group). Different types of bariatric surgery included a 46.88% of RYGB, 33.76% laparoscopic sleeve gastrectomy (LSG), 10.66% adjustable gastric banding (AGB), and 8.69% others. After propensity-score matching, the final study cohort included 537,757 patients with bariatric surgery and 537,757 patients with other abdominal surgeries. (Fig. 1). All analyses were performed in this final matched cohort. The two groups had similar baseline demographic characteristics and comorbidities (Table 1). The two groups (bariatric surgery vs abdominal surgery, respectively) were comparable with homogeneous proportion of patients having metabolic comorbidities, including diabetes (18.5% vs 19.2%), hypertension (43.4% vs 46.3%), obesity (16.9% vs 19.3%), and renal failure (4.3% vs 5.3%). The prevalence of vitamin D deficiency was significantly higher in the bariatric surgery group (2.41% vs 0.48%; OR: 5.12 [95% CI: 4.91–5.34]; *p* < 0.001) (Fig. 2S).

**Table 1:** Baseline characteristics of hospitals admissions in matched patients with previous bariatric surgery vs abdominal surgery^†^

	Bariatric surgery (*n* = 537,757)	Abdominal surgery (*n* = 537,757)	Std. diff (%)
Baseline characteristics			
Gender, M/F (%)	143,365(26.7)/394,392(73.3)	149,171(27.7)/388,586 (72.3)	2.4
Age (yr)	48.78 ± 15	51.51 ± 18	16.3
Ethnic origin (%)			
Caucasian	389,159 (72.4)	380,259 (70.7)	3.8
Af. American	66,960 (12.5)	71,044 (13.2)	
Hispanic	56,525 (10.5)	58,908 (11.0)	
Others	25,113 (4.7)	27,546 (5.1)	
Primary expected payer (%)			
Medicare	140,423 (26.1)	153,868 (28.6)	7.6
Medicaid	65,504 (12.2)	67,650 (12.6)	
Private	284,424 (52.9)	264,819 (49.2)	
Self-pay	26,260 (4.9)	29,211 (5.4)	
Median household income national quartile for patient ZIP code (%)			
1	124,661 (23.7)	141,065 (26.8)	7.3
2	134,429 (25.6)	132,421 (25.1)	
3	138,664 (26.4)	130,878 (24.9)	
4	127,975 (24.3)	122,265 (23.2)	
AHRQ comorbidity measure			
Deficiency anemias (%)	69,401 (12.9)	73,837 (13.7)	5.2
Rheumatoid (arthritis/collagen vascular) (%)	12,385 (2.3)	10,949 (2.0)	2.4
Chronic blood loss anemia (%)	8,306 (1.5)	5,021 (0.9)	1.8
Congestive heart failure (%)	16,914 (3.1)	25,178 (4.7)	5.5
Chronic pulmonary disease (%)	90,034 (16.7)	75,355 (14.0)	7.9
Coagulopath (%)	13,912 (2.6)	15,224 (2.8)	1.5
Diabetes, uncomplicated (%)	99,618 (18.5)	103,409 (19.2)	1.8
Diabetes (with chronic complications) (%)	12,420 (2.3)	13,995 (2.6)	1.9
Hypertension (%)	233,376 (43.4)	248,876 (46.3)	5.8
Hypothyroidism (%)	66,099 (12.3)	52,016 (9.7)	8.4
Liver disease (%)	31,394 (5.8)	25,603 (4.8)	4.8
Lymphoma (%)	1,500 (0.)	1,884 (0.4)	1.3
Fluid and electrolyte disorders (%)	82,482 (15.3)	104,101 (19.4)	10.6
Metastatic cancer (%)	8,985 (1.7)	10,557 (2.0)	2.2
Other neurological disorders (%)	25,597 (4.8)	20,681 (3.8)	4.5
Obesity (%)	90,672 (16.9)	103,714 (19.3)	6.3
Paralysis (%)	5,226 (1.0)	5,567 (1.0)	0.6
Peripheral vascular disorders (%)	9,584 (1.8)	15,002 (2.8)	6.7
Psychoses (%)	29,146 (5.4)	15,377 (2.9)	12.9
Pulmonary circulation disorders (%)	5,697 (1.1)	6,671 (1.2)	1.7
Renal failure (%)	22,944 (4.3)	28,546 (5.3)	4.9
Solid tumor without metastasis (%)	4,091 (0.8)	6,183 (1.1)	4.0
Peptic ulcer disease (excluding bleeding) (%)	1,113 (0.2)	214 (0.0)	4.8
Valvular disease (%)	9,679 (1.8)	16,017 (3.0)	7.7
Weight loss (%)	24,581 (4.6)	25,983 (4.8)	1.2

Data are presented as mean ± standard deviation or frequencies (%)

Abbreviations: *AHRQ* agency for healthcare research and quality, *F* female, *M* male, *Yr* years, *Af* African American, *Std. diff* standard differences

^†^Study groups matched: within the bariatric surgery group, all the patients with previous bariatric surgery. Within the abdominal surgery group, all the patients with other abdominal surgeries according to ICD-9 codes

### Alcohol Use Disorder

Patients in the bariatric surgery group had a significantly increased risk of AUD (3.32% vs 1.74%; OR: 1.90 [95% CI: 1.85–1.95]; *p* < 0.001). In the bariatric surgery group, the prevalence of AUD increased significantly from 2005 to 2015 (Fig. 2). The magnitude of the association between AUD and bariatric surgery was stronger in patients without vitamin D deficiency (OR 2.48 in normal vitamin D group vs OR 1.27 in vitamin D deficiency group).

### Alcohol-Related Liver Disease

**Fig. 3:**
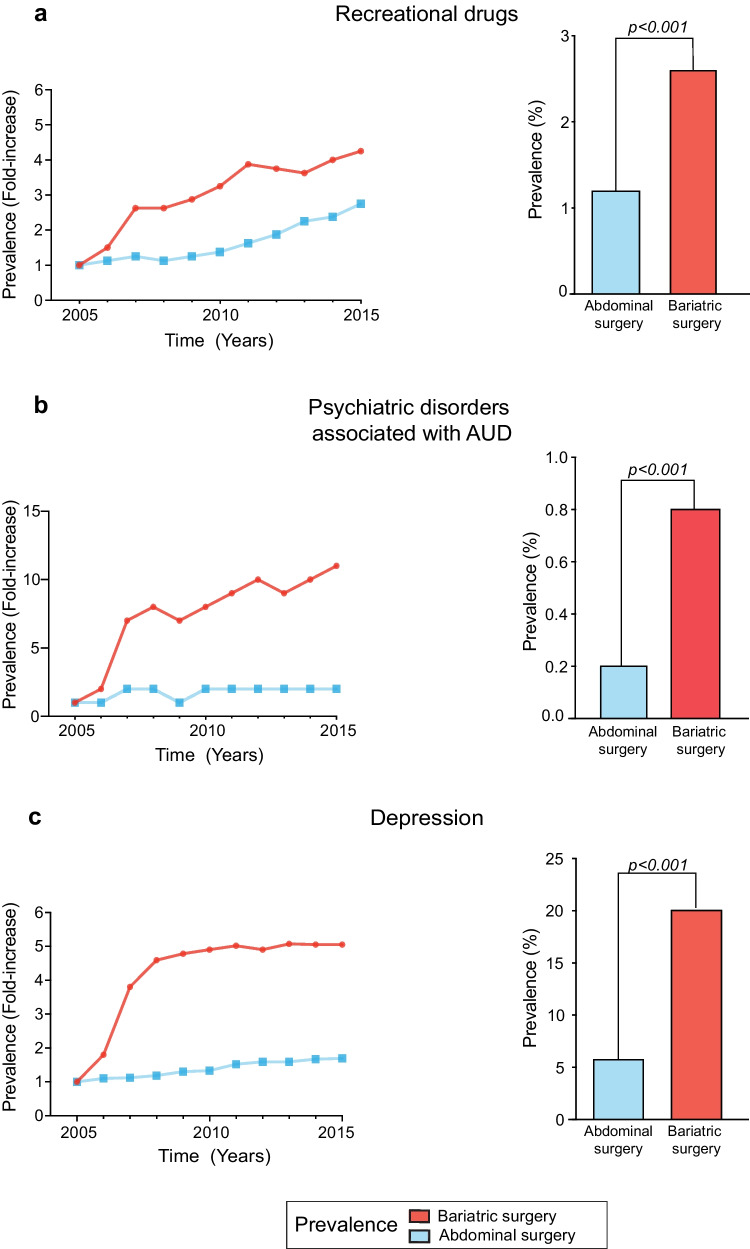
Impact of bariatric surgery on the incidence of psychiatric disorders associated with AUD. Results are expressed as annual fold changes (left side) and overall prevalence rate (right side) during the study period (2005 to 2015). **a** Prevalence of recreational drugs use; **b** prevalence of psychiatric disorders associated with AUD; and **c** prevalence of depression in the bariatric surgery and abdominal surgery groups

**Fig. 4:**
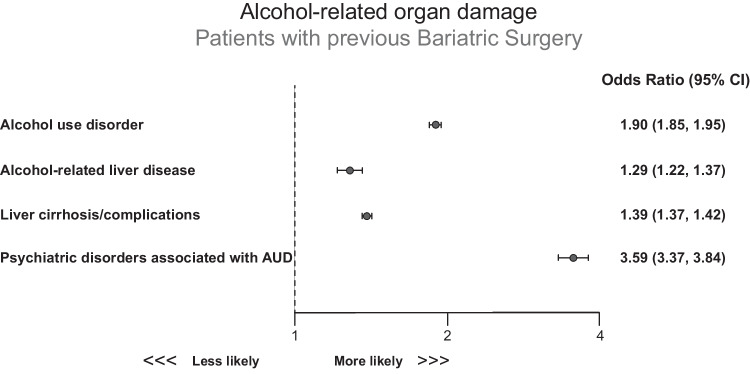
Alcohol-related organ damage in the bariatric surgery group. Results are expressed as odds ratios (OR) with 95% confidence intervals (CI). The OR were calculated using conditional stratified logistic regression accounting for matched pairs as random effects

The prevalence of ALD was significantly higher in the bariatric surgery group (Table 2). Compared to abdominal surgery, bariatric group had a greater risk of ALD (0.71% vs 0.43%, OR: 1.29 [95% CI: 1.22–1.37]; *p* < 0.001). Among patients with AUD, the rate of ALD was higher after bariatric surgery than other abdominal surgeries (3.4% vs 1.4%; OR: 2.47 [95% CI: 2.45–2.50]; *p* < 0.001). The prevalence of cirrhosis was also significantly higher in the bariatric surgery group (5.34% vs 3.92%, OR: 1.39 [95% CI: 1.37–1.42]; *p* < 0.001). The prevalence of both ALD and cirrhosis showed a significant increase among bariatric surgery cases from 2005 to 2015 (Fig. 2). The main etiology of liver disease associated to bariatric surgery was nonalcoholic fatty liver disease followed by ALD, with a 4.4% and 0.7% prevalence, respectively (Fig. 3S). The prevalence of liver disease differed according to vitamin D status (Fig. 4S). Among patients with vitamin D deficiency, ALD prevalence was similar between surgical groups (Table 3).

**Table 2:** Alcohol-related organ damage in both groups matched, with bariatric surgery vs abdominal surgery^†^

	Bariatric surgery (*n* = 537,757)	Abdominal surgery (*n* = 537,757)	*p* value
Alcohol use disorder, (%)	17,853 (3.32)	9,359 (1.74)	< 0.001
Alcohol-related liver disease, (%)	3,818 (0.71)	2,335 (0.43)	< 0.001
Liver cirrhosis/complications, (%)	28,716 (5.34)	21,080 (3.92)	< 0.001
Psychiatric disorders associated with AUD, (%)	4,180 (0.80)	1,169 (0.21)	< 0.001
Recreational drugs use, (%)	13,981 (2.6)	6,453 (1.2)	< 0.001
Depression, (%)	108,089 (20.1)	31,189 (5.8)	< 0.001

Data are presented as frequencies (%)

^†^Study groups not matched: Within the bariatric surgery group, all the patients with previous bariatric surgery. Within the abdominal surgery group, all the patients with other abdominal surgeries according to ICD-9 codes

**Table 3:** Alcohol-related diseases in the matched cohort, stratified by vitamin D status in bariatric surgery group vs abdominal surgery group

Vitamin D deficiency				
Clinical outcome	Bariatric surgery (*n* = 17,677)	Abdominal surgery (*n* = 3,562)	OR (95% CI)	*p* value
Alcohol use disorder	2.90%	2.30%	1.27 (1.02–1.61)	0.04
Alcohol-related liver disease	0.40%	0.31%	1.25 (0.66–1.37)	0.40
Liver cirrhosis/complications	10.5%	7.0%	1.56 (1.36–1.78)	< 0.001
Psychiatric disorders associated with AUD	0.5%	0.1%	3.88 (1.58–9.55)	0.003
Vitamin D normal				
Clinical outcome	Bariatric surgery (*n* = 729,202)	Abdominal surgery (*n* = 1,447,148)	OR (95% CI)	*p* value
Alcohol use disorder	3.40%	1.40%	2.48 (2.44–2.53)	< 0.001
Alcohol-related liver disease	0.61%	0.10%	3.90 (3.70–4.11)	< 0.001
Liver cirrhosis/complications	5.20%	2.50%	2.09 (2.06–2.12)	< 0.001
Psychiatric disorders associated with AUD	0.80%	0.21%	4.79 (4.57–5.02)	< 0.001

Data are presented as frequencies (%). Odds ratio (OR) (95%, confidential interval) and *p* value

### Psychiatric Disorders Associated with AUD

The prevalence of psychiatric disorders associated with AUD, recreational drug use, and depression was higher in the bariatric surgery group (Fig. 3). Compared to abdominal surgery, bariatric procedures had a higher risk of developing psychiatric disorders associated with AUD (Table 2). Bariatric surgery was more strongly associated with psychiatric disorders in AUD (OR: 3.59 [95% CI: 3.37–3.84]) than with alcohol-related liver disease (OR: 1.29 [95% CI: 1.22–1.37]) (Fig. 4). Psychiatric disorders in AUD and bariatric surgery association were similar regardless of vitamin D status (Table 3).

## Discussion

This study determined whether bariatric surgery was associated with the development of liver and mental disorders, and the role—if any—of vitamin D deficiency. These findings show that patients who underwent bariatric surgery had a significantly increased risk of developing AUD, ALD, cirrhosis, and psychiatric disorders associated with AUD. Vitamin D deficiency did not appear to influence the association between bariatric surgery and AUD, ALD, or psychiatric disorders associated with AUD.

The motivation to perform the present study was based on clinical observations in the routine practice, in which some patients who had previously undergone bariatric surgery developed ALD, including alcoholic hepatitis. Although bariatric surgery has been shown to have beneficial effects in patients with nonalcoholic steatohepatitis, long-term outcomes need to be interpreted with caution [28–30]. Moreover, given that many clinicians are not aware that bariatric surgery may predispose patients to AUD, it was considered necessary to study the relationship between metabolic surgery and alcohol-related outcomes, such as liver diseases and psychiatric disorders associated with AUD.

The data for this study was obtained from the NIS database. Other studies have also used this database, as to demonstrate an increasing prevalence of ALD cirrhosis in the U.S [31–33]. Diagnostic and comorbidity data from all hospital discharges between 2005 and 2015 was analyzed. The outcomes being studied were excluded prior to surgery. Given that patients undergoing bariatric surgery are considered to have a major risk factor for liver disease, a propensity match was also performed prior to surgery to reduce the impact of confounding variables. This design allowed for the comparison between groups and to establish risk associations.

Our data show that AUD was more prevalent among patients who have undergone bariatric surgery, confirming previous experimental and clinical studies [1, 2, 5, 34]. The association between bariatric surgery and AUD was previously assessed in the LABS-2 (Longitudinal Assessment of Bariatric Surgery-2) prospective cohort studies, which showed a cumulative incidence of AUD symptoms of 10–21% in the 2–5 years period after surgery [1, 2]. Our results are similar, although the prevalence of AUD was lower. Different prevalences may be due to several factors: first, the underreporting of psychiatric diagnoses in coding-based medical record databases. Our study highlights this major issue that should alert clinicians to evaluate AUD in bariatric surgery patients. Second, previous studies determined AUD symptoms with the Alcohol Use Disorders Identification Test (AUDIT) which is more specific. Third, previous studies included preoperative AUD cases with a prevalence of 8–10% [1, 35]. Our study did not include preoperative AUD cases, showing a similar fold increase in postoperative AUD prevalence. Nevertheless, beyond coding and AUDIT evaluation, objective alcohol biomarkers such as phosphatidylethanol in whole blood or ethyl glucuronide in hair specimens are needed to establish the true prevalence of AUD in this population.

Several mechanisms have been suggested to explain the association between bariatric surgery and AUD: (1) changes in alcohol pharmacokinetic profile, with an earlier and higher peak of BAC mainly in RYGB, disproportionately high compared to the dose consumed [10–12, 34]; (2) changes in the taxonomic profile of the gut microbiota after bariatric surgery, particularly RYGB, are associated with an increased alcohol intake and may contribute to increased AUD risk through the gut-brain-microbiome axis [36]; and (3) neuro-hormonal shifts after RYGB that determine changes in reward processing [14, 15, 17, 18, 37, 38]. Considering these mechanisms, related organ damage is likely from a high but quickly resolving peak of BAC. Our data support the need for more clinical and basic research in this area to better elucidate which of these postulated mechanisms have the greatest impact. Such studies would also be useful to identify ways to target or prevent AUD in patients undergoing bariatric surgery.

In this study, it was also found that ALD and liver cirrhosis were more prevalent in the bariatric surgery group, with a trend towards significant annual increases in the ten-year study period (Fig. 2). These results are consistent with the recent findings of a study performed to assess the potential long-term increased risk of alcohol-related cirrhosis in women who underwent bariatric surgery [4, 24]. Although statistically significant, the bariatric surgery group had a low prevalence of ALD (0.7%). Within selected patients with AUD, ALD prevalence raised up to 3.4%. Despite of these low prevalences, the clinical significance of our findings from a large sample database relies on the 29% increased risk of developing ALD (OR: 1.29) after bariatric surgery in comparison to other abdominal surgeries. To differentiate obesity-associated liver disease from AUD-associated liver disease, different ICD codes were used to define these conditions, and a propensity score analysis was performed considering obesity as a confounding variable. The overall prevalence of liver disease in bariatric surgery patients was 4.4% and 0.7% for nonalcoholic fatty liver disease and ALD, respectively.

Despite a higher risk of mental disorders among patients undergoing bariatric surgery, the current study takes a step further and focuses on psychiatric disorders associated with AUD. Beyond recreational drug use and depression, psychiatric disorders associated with AUD were also more prevalent in the bariatric surgery group. Although psychiatric disorder and alcohol abuse developed after surgery, it is not possible to know which of the two disorders led to the other one [25]. One of the mechanisms underlying these comorbidities could be modifications in the pharmacokinetics of alcohol [11, 34], which generates greater susceptibility to its effects, thus leading to alcohol-mediated organ damage. Given the weight loss associated with bariatric surgery, patients who drink will experience an increased tissue dose postoperatively, even if they do not increase their alcohol intake. In the study group was observed an increase in the prevalence of the main outcome variables (AUD, ALD, psychiatric disorders associated with AUD over the 10-year study period), which could be partially explained by the greater awareness of alcohol-related diseases and nutritional deficiencies in recent years. Increased awareness of these conditions may have led to an increase in the diagnosis and subsequent ICD coding for these disorders.

Another mechanism that has been proposed to explain the association between bariatric surgery and alcohol-related liver diseases is vitamin D deficiency [39–41]. However, in this study, the association between bariatric surgery and ALD seemed to be independent from vitamin D deficiency, indicating that other mechanisms besides nutritional deficiencies may be involved.

The findings of this study, based on a large national dataset, have a practical clinical application. This is the need to increase awareness about the association between bariatric surgery and alcohol-related issues and thus the need for prevention and education, as well as the monitoring of alcohol use and liver injury before and after bariatric procedures.

The current study has several limitations. First, due to the cross-sectional design, it is possible to identify associations but not to test for causality. Second, the data source has a limited capacity to verify the quality of the diagnostic and comorbidity coding at hospital discharge. Third, bariatric surgery included procedures with different reported risks for new onset AUD in previously published articles [1, 2, 35]. The most frequent procedures were RYGB and LSG (around 80%) which have shown a higher risk of AUD compared to AGB [1, 2, 35]. Procedures other than RYGB and LSG were included because the prevalence of AUD post-AGB is non-neglectable (up to 11% in the LABS-2 cohort) [2], and they represented a minor proportion of the surgical procedures from the NIS database. Still, a risk association between AUD and bariatric surgery was seen, although interpretation should be mainly subject to RYGB and LSG procedures. Further studies should evaluate whether these different interventions differ in terms of incident ALD and psychiatric disorders associated with AUD, among others. Fourth, AUD and psychiatric disorders associated with AUD can be under-reported and underrecognized, particularly in patients being treated for medical conditions unrelated to alcohol use, and especially in databases based on ICD codes [42]. Finally, the analysis was performed until 2015 because the clinical terms used corresponded to ICD-9 codes. At the end of 2015, codification was updated from ICD-9 to ICD-10 codes, resulting in the unavailability of previous databases.

Notwithstanding the potential limitations described above, our data show that the prevalence of AUD, ALD, and psychiatric disorders associated with AUD was higher in patients who had undergone bariatric surgery than other abdominal surgeries. Although prospective studies are needed to better determine alcohol use and whether there is a causal relationship between metabolic surgery and alcohol-related diseases, our results underscore the importance of counseling for alcohol-associated disorders in patients undergoing bariatric surgery. In this regard, all candidates for bariatric surgery should undergo in-depth clinical interviews to detect patterns of alcohol use and risk factors for AUD, including a comprehensive assessment of specific alcohol biomarkers (such as phosphatidylethanol or ethyl glucuronide) with blood, urine, or hair samples analysis before and after bariatric surgery [43, 44]. Prospective studies are needed to evaluate long-term outcomes in this patient population. Ideally, these studies would include regular clinical evaluations with biospecimen collection performed at baseline and annually thereafter. This study design would allow us to establish causality between bariatric surgery and the subsequent development of alcohol-related conditions. In addition, it would help to better elucidate the molecular mechanisms underlying these associations and to identify high-risk patients.

In summary, our data suggest that the evident benefits of bariatric surgery must be weighed against the risks of developing AUD, which can lead to ALD and/or psychiatric disorders associated with AUD. It is especially important to exercise caution before performing bariatric surgery in patients who present advanced liver disease and/or alcohol misuse [45]. Finally, although our data indicate that vitamin D deficiency does not appear to be associated with the risks of AUD, ALD, or psychiatric disorders associated with AUD, further studies should explore other potential mechanisms underlying the development of these conditions after bariatric surgery.

## Supplementary Information

Below is the link to the electronic supplementary material.

Supplementary file1 (PDF 297 KB)

## Abbreviations

ALD: Alcohol-related liver disease
AUD: Alcohol use disorder
AMD: Alcohol-related mental disorder
BMI: Body mass index
CI: Confidential interval
GLP-1: Glucagon-like peptide-1
ICD: International Classification of Diseases
LAGB: Laparoscopic adjustable gastric banding
NNIDDK: National Institute of Diabetes, Digestive and Kidney Diseases
LAB-2 Study: NIDDK-funded Longitudinal Assessment of Bariatric Surgery-2
NIS: National Inpatient Sample
OR: Odds ratio
RYGB: Roux-en-Y gastric bypass
SD: Standard deviation
SG: Sleeve gastrectomy
