# Supplementary material for: Bariatric Surgery Is Associated with Alcohol-Related Liver Disease and Psychiatric Disorders Associated with AUD
Source: Obes Surg. 2023 Mar 7;33(5):1494–505. doi: 10.1007/s11695-023-06490-w (PMC10156826; doi:10.1007/s11695-023-06490-w)
Supplement: Supplementary file 1 — Supplementary file1 (PDF 297 KB) [file 11695_2023_6490_MOESM1_ESM.pdf]

BARIATRIC SURGERY IS ASSOCIATED WITH ALCOHOL-RELATED LIVER DISEASE AND PSYCHIATRIC DISORDERS ASSOCIATED WITH AUD (Reviewer 2, comment 02).

SUPPLEMENTARY MATERIAL

Figure 1S. Graphical summary of the study design.

Finally selected population, main outcomes, and variables obtained from hospital discharges information between the years 2005-2015.

Figure 2S. Impact of bariatric surgery on the development of vitamin D deficiency.

Results are expressed as annual fold-change increases from baseline and the overall prevalence (%) of vitamin D deficiency from 2005 to 2015 in the bariatric surgery *vs* the abdominal surgery group.

Figure 3S. Impact of bariatric surgery on liver disease of different etiologies

Results are expressed as frequencies (%). Prevalence of the main 2 etiologies. Abbreviations: ALD, Alcohol-related liver disease; NAFLD, Non-alcoholic fatty liver disease.

Figure 4S. Impact of vitamin D deficiency on the development of alcohol-related organ damage in the bariatric surgery group.

Data are expressed as frequencies (%) according to vitamin D status. **(a)** Prevalence of alcohol use disorder. **(b)** Prevalence of alcohol-related liver disease. **(c)** Prevalence of cirrhosis. **(d)** Prevalence of psychiatric disorders associated with AUD (Reviewer 2, comment 02).

TABLE 1S. ICD-9 codes used for cohort selection and comorbidities, as well as excluded diagnoses.

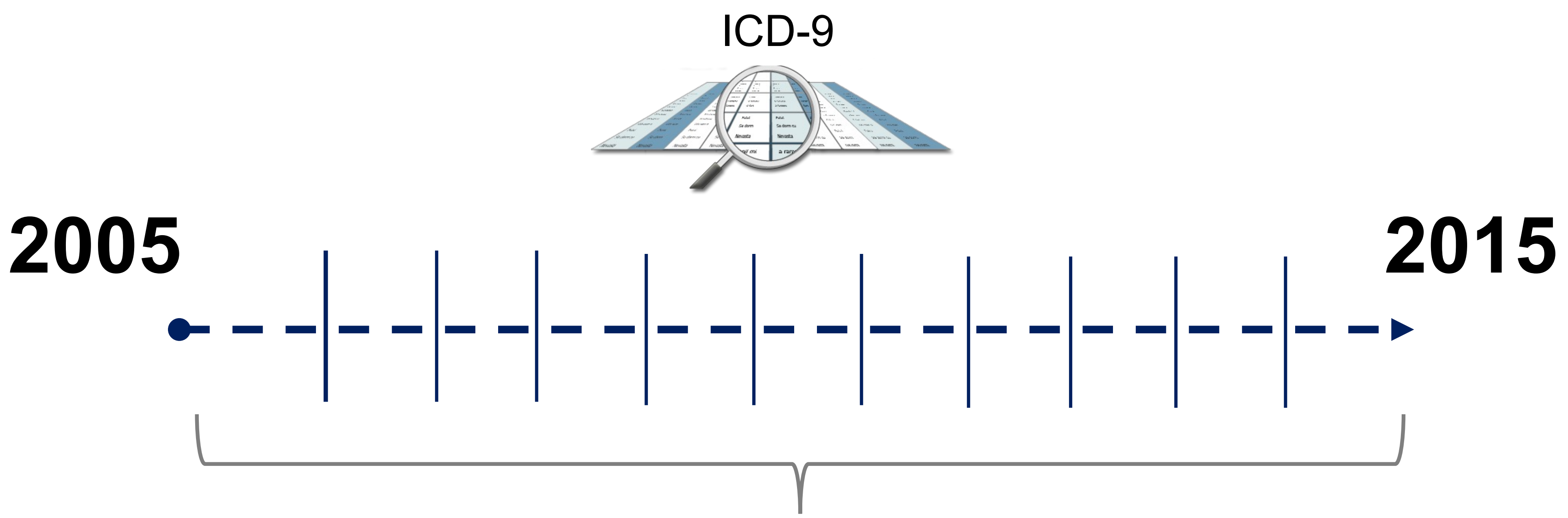

- **Design**: yearly cross-sectional observational study using NIS database and ICD-9 codes
- **Inclusion**: hospital discharges with history of bariatric or abdominal surgery
- **Exclusion**: cases with the outcomes of interest present prior to surgery
- **Evaluation**: propensity score matching performed prior to surgery
- **Outcomes of interest**: AUD, ALD, psychiatric disorders associated with AUD

Fig. 1S

Vitamin D Deficiency

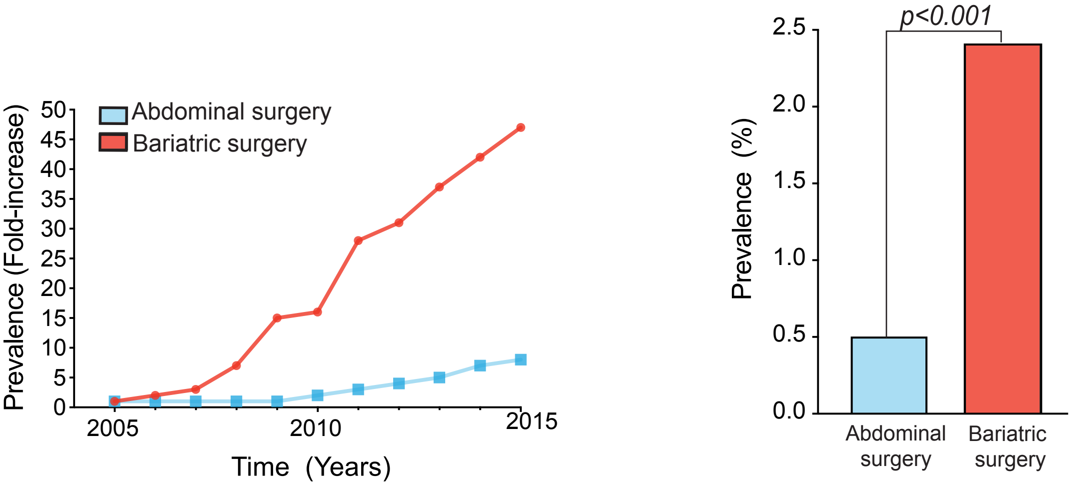

Fig. 2S

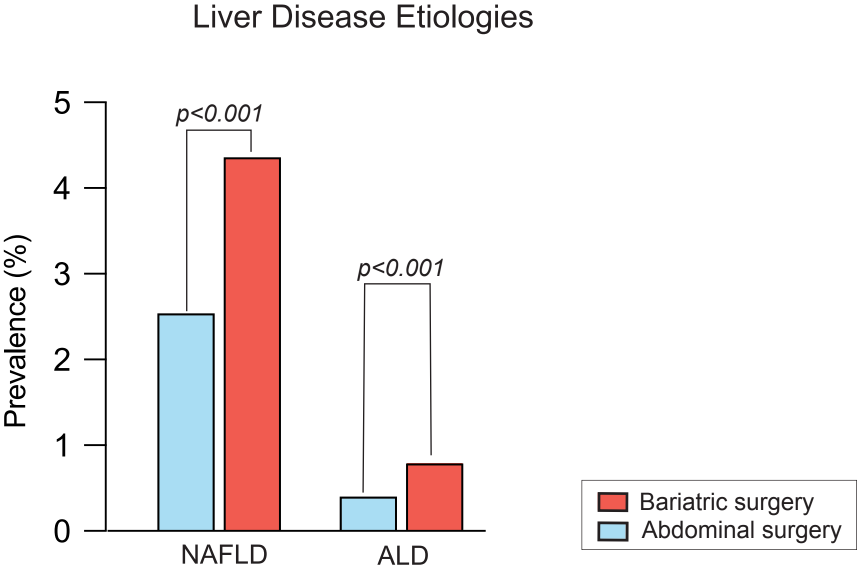

Liver Disease Etiologies
5
4
3
2
1
0
Prevalence (%)
p<0.001
p<0.001
NAFLD
ALD
Bariatric surgery
Abdominal surgery

Fig. 3S

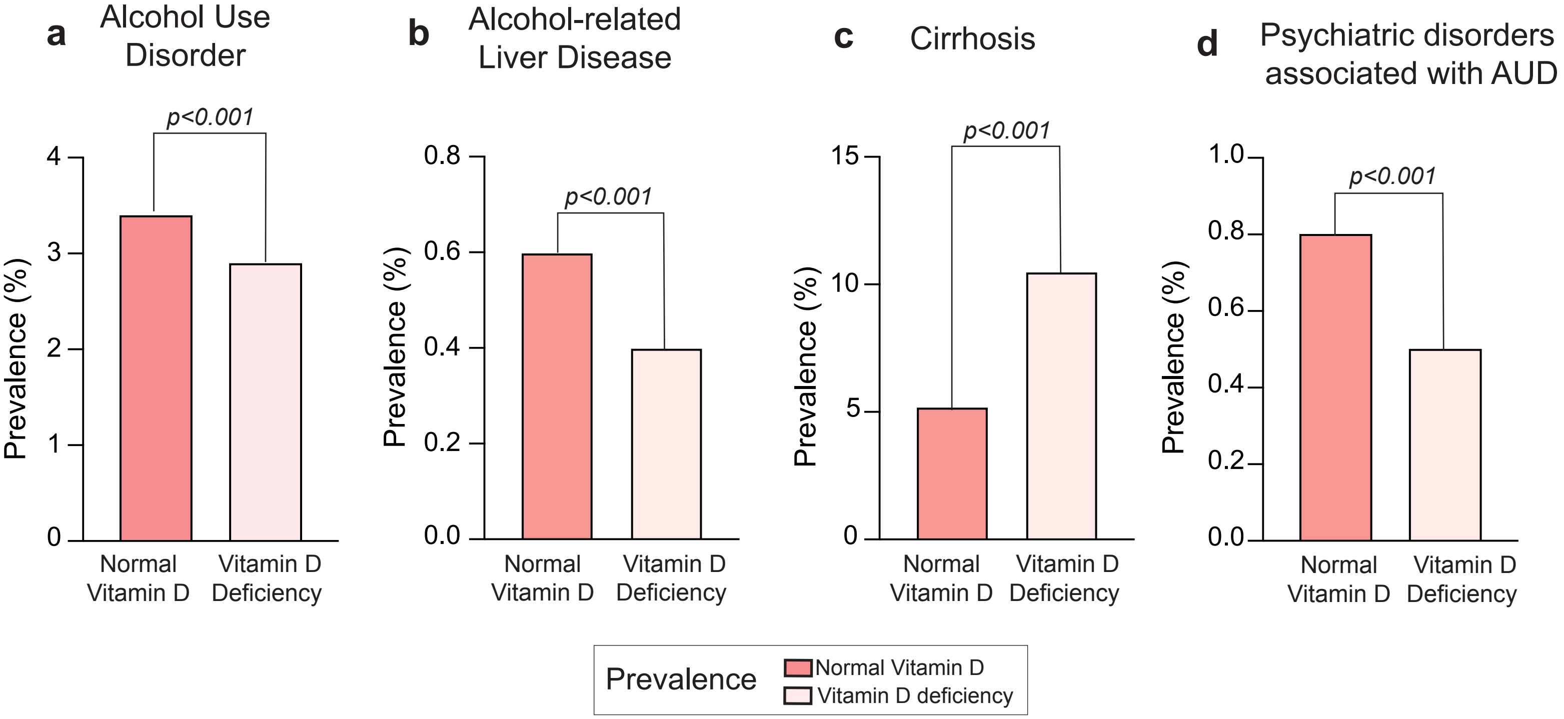
a
Alcohol Use Disorder
p<0.001
Prevalence (%)
0
1
2
3
4
Normal Vitamin D
Vitamin D Deficiency
b
Alcohol-related Liver Disease
p<0.001
Prevalence (%)
0.0
0.2
0.4
0.6
0.8
Normal Vitamin D
Vitamin D Deficiency
c
Cirrhosis
p<0.001
Prevalence (%)
0
5
10
15
Normal Vitamin D
Vitamin D Deficiency
d
Psychiatric disorders associated with AUD
p<0.001
Prevalence (%)
0.0
0.2
0.4
0.6
0.8
1.0
Normal Vitamin D
Vitamin D Deficiency
Prevalence
Normal Vitamin D
Vitamin D deficiency

Fig. 4S

TABLE 1S. ICD-9 codes used for cohort selection and comorbidities, as well as excluded diagnostics.

DIAGNOSIS	ICD-9 CODES
**BARIATRIC SURGERY**	
Bariatric surgery status	V45.86
Fitting and adjustment of gastric lap	V53.51
Complications of bariatric surgery	539.X
Gastric restrictive procedure	44.69
Laparoscopic vertical (sleeve) gastrectomy	43.82, 43.89
Laparoscopic adjustable gastric band	44.95
Replacement/adjustment of gastric band	44.96,44.98
Gastric bypass	44.32
Malabsorption (gastric bypass)	579,3
Partial gastrectomy with bypass gastro-gastrostomy	43.89
High gastric bypass	44.31
Laparoscopic gastric bypass with small intestine reconstruction to limit absorption	44.38,44.39
Open Roux-en-Y gastric bypass	44.39, 44.31
**ABDOMINAL SURGERIES**	
Appendectomy	47.0
Incidental appendectomy	47.1
Laparoscopic appendectomy	47.01
Laparoscopic incidental appendectomy	47.1
Cholecystectomy	51.22
Laparoscopic cholecystectomy	51.23
**ALCOHOL**	
**Alcohol use disorder.**	
Alcohol dependence syndrome	303.x (0/00/01/02/03/9/90/91/92/93)
Acute alcoholic intoxication in alcoholism, unspecified	303,00
Acute alcoholic intoxication in alcoholism, continuous	303,01
Other and unspecified alcohol dependence, continuous	303,91
Alcohol abuse, unspecified	305.0X
Alcohol abuse, continuous	305,01
Personal history of alcoholism	V11.3
Alcohol use, unspecified	790.3, 425.5, 535.30,535.51,577,357.5, 291.x, 303.x,305.0x, V113.0, E860.01-03,980.
**Alcohol - related Liver disease**	
Fatty liver, alcoholic	571
Acute Alcoholic Hepatitis	571.1
Cirrhosis of the liver with alcoholism	571.2
Alcoholic Liver Damage, Unspecified	571.3
Alcoholic liver disease	571.1,571.2,571.3
**Liver Cirrhosis/Decompensation**	
Alcoholic cirrhosis of liver	571.2
Chronic Liver disease and cirrhosis	571/571.5
Portal Hypertension	572.3
Hepatic encephalopathy	572.2
Esophageal varices with bleeding	456.0, 456.2
Esophageal varices without mention of bleeding	456.1
Esophageal varices in diseases classified elsewhere with bleeding	456.21

Esophageal varices in diseases classified elsewhere without mention of bleeding	456.21
Spontaneous bacterial peritonitis	567.23
Hepatorenal syndrome	572.4
Hepatopulmonary syndrome	573.5
Ascites	789.5
**Psychiatric disorders associated with AUD**	
Alcohol-induced psychotic disorder	291.X
Alcohol abuse with Depressive disorders	296.21-296.36
Alcohol abuse with bipolar-unspecified	296,8
Alcohol abuse with anxiety state-unspecified	300.0
Alcohol abuse with panic disorder	300.01
Alcohol abuse with stress-unspecified	308.9
Alcohol abuse with history of emotional abuse	V15.42
**EXCLUSION CRITERIA**	
Malignant neoplasm of esophagus	150.x
Malignant neoplasm of stomach	151.x
Malignant neoplasm of small intestine, including duodenum	152.x
Malignant neoplasm of pancreas	157.x
Gastric ulcer	531.x
Duodenal ulcer	532.x
Peptic ulcer, sit unspecified	533.x
Malignant neoplasm of esophagus	150.x
Children and pregnancy	V22.1

Abbreviations: ICD, International Classification of Diseases.
